# Retrospective evaluation of nimustine use in the treatment of feline lymphoma

**DOI:** 10.1002/vms3.652

**Published:** 2021-10-02

**Authors:** Kosei Sakai, Shingo Hatoya, Masaru Furuya, Tomoyo Nabetani, Ryoji Kanegi, Shunsuke Shimamura, Hiroyuki Tani, Terumasa Shimada

**Affiliations:** ^1^ Veterinary Medical Centre, Graduate School of Life and Environmental Sciences Osaka Prefecture University Osaka Japan

**Keywords:** adverse events, cats, clinical outcomes, lymphoma, nimustine

## Abstract

**Background:**

Nimustine, similar to lomustine, is an alkylating agent from the nitrosourea family. There have been some reports regarding lomustine treatment for tumour‐bearing cats. However, information regarding nimustine treatment for tumour‐bearing cats is limited.

**Objectives:**

To retrospectively evaluate adverse events and clinical outcomes in tumour‐bearing cats receiving nimustine.

**Methods:**

Information regarding diagnosis, treatment condition, adverse events, and clinical outcomes was collected in tumour‐bearing cats receiving nimustine through reviews of medical records.

**Results:**

Nine cats with lymphoma were treated with nimustine in the primary therapy (*n* = 2) and in the rescue therapy (*n* = 7). Median starting dose of nimustine was 25 mg/m^2^ (range: 20–30 mg/m^2^) with dosing interval of three weeks and 1–11 administrations. Adverse events were mild gastrointestinal toxicity (grade 1) including diarrhoea (*n* = 2) and vomiting (*n* = 2) and mild myelosuppression (grade 1 or 2) including thrombocytopenia (*n* = 3) and neutropenia (*n* = 1). No severe adverse events were observed. Progression‐free survival durations among cats receiving nimustine in the primary therapy and in the rescue therapy were 274–688 days (median: 481 days) and 9–671 days (median: 102 days), respectively. Overall survival durations among cats receiving nimustine in the primary therapy and in the rescue therapy were 275–745 days (median: 510 days) and 14–671 days (median: 109 days), respectively.

**Conclusions:**

Nimustine was well tolerated and showed clinical outcomes similar to lomustine in cats with lymphoma. These findings suggest that nimustine might be an alternative to lomustine in the treatment of feline lymphoma.

## Graphical abstract

This study was retrospectively evaluate adverse events and clinical outcomes in cats receiving nimustine. Nimustine was well tolerated and showed clinical outcomes similar to lomustine in cats with lymphoma, suggesting that nimustine might be an alternative to lomustine in the treatment of feline lymphoma.

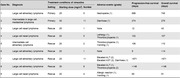

Lomustine is an alkylating agent belonging to the nitrosourea family, which damages DNA. This agent is highly lipid‐soluble (Oliverio, 1973). Thus, it is administered as an oral capsule. Lomustine has been shown to be effective against feline lymphoma and mast cell tumour (Dutelle et al., 2012; Rassnick et al., 2001; Rassnick et al., 2008; Rau & Burgess, 2017).

Similar to lomustine, nimustine is also an alkylating agent belonging to the nitrosourea family. In contrast to lomustine, nimustine is water‐soluble. Thus, it is administered as an injectable agent. Takahashi et al. (2014) conducted a phase I study of nimustine in dogs with various tumours. They demonstrated acceptable safety and adequate tolerability of nimustine in dogs at a dose of 25 mg/m^2^ with dosing interval of three weeks. Nimustine‐associated adverse effects in dogs were myelosuppression and gastrointestinal toxicity. Moreover, a recent study has reported that nimustine shows outcomes similar to those observed with lomustine in terms of progression‐free survival (PFS) and overall survival (OS) in canine histiocytic sarcoma (Tani et al., 2020). A case report showed that long‐term remission was achieved in a cat with anaplastic oligodendroglioma treated with radiation therapy followed by nimustine (Tamura et al., 2013). However, information regarding safety and efficacy of nimustine treatment in cats is limited. Therefore, the purpose of this study was to retrospectively evaluate the adverse events and clinical outcomes in tumour‐bearing cats receiving nimustine.

Medical records of the Veterinary Medical Centre of Osaka Prefecture University were investigated for tumour‐bearing cats receiving at least one dose of nimustine (Nidran; Daiichi Sankyo, Tokyo, Japan) from May 2016 to August 2020. The following data were collected from each case for the analysis: signalment (age, sex, neutered status, breed, infection status of feline leukaemia virus [FeLV] and feline immunodeficiency virus [FIV], and concomitant diseases), diagnosis, diagnostic methods, clinical stage, treatments prior to nimustine, treatment condition of nimustine, treatments after nimustine, adverse events during nimustine treatment, and clinical outcomes.

To assess clinical response and adverse events in the cats receiving nimustine, interview of clinical signs to owners, physical examination, blood tests (complete blood count and blood chemistry), and/or imaging examination were performed at visits. Since it was difficult to observe lesions in many cases by radiography and ultrasonography, objective evaluation (complete remission, partial remission, stable disease, and progressive disease) was not determined in this study. Tumour progression was defined as appearance or worsening of tumour‐associated clinical signs, local progression, local recurrence, or metastasis. Adverse events were graded according to the Veterinary Comparative Oncology Group Common Terminology Criteria (Veterinary Co‐operative group, 2016).

The status of each cat (alive, dead, or lost) until the end of the study (3 December 2020) was obtained from medical records. PFS was defined as the duration from the initial nimustine administration to tumour progression or death at the end of the study. OS was defined as the interval between the initial nimustine administration and the established cause of death at the end of the study. The Kaplan–Meier method was used to calculate median PFS and OS. Statistical analysis was performed using GraphPad Prism software version 8.3.0 (Graph Pad Software Inc., San Diego, California).

Nine cats were included in this study (Table 1). Median age was 122 months (range: 12–203 months) at the time of initial nimustine administration. The cats included in the study comprised of one intact male, four castrated males, and four spayed females, and there were of different breeds. Infections with FeLV or FIV were detected in one cat each. The remaining seven cats did not have any concomitant diseases based on physical examination, complete blood count, blood chemistry test, chest and abdominal radiography, and abdominal ultrasonography.

**TABLE 1:** Profile of cats with lymphoma used in this study

Case No.	BW (kg)	Age (months)	Sex	Neutered status	Breed	FeLV	FIV
1	4.1	12	Female	Spayed	Mixed	+	–
2	4.4	177	Male	Castrated	Ragdoll	–	–
3	2.8	122	Female	Spayed	Mixed	–	–
4	2.9	55	Male	Castrated	Chinchilla	–	–
5	3.3	129	Male	Castrated	Scottish Fold	–	–
6	1.7	203	Female	Spayed	Russian Blue	–	+
7	3.8	121	Male	Intact	Scottish Fold	–	–
8	4.7	83	Male	Castrated	Mixed	–	–
9	4.3	127	Female	Spayed	Mixed	–	–

Abbreviation: BW, body weight; FeLV, feline leukaemia virus; FIV, feline immunodeficiency virus.

As shown in Table 2, all cats were diagnosed with lymphoma including alimentary lymphoma (*n* = 5), nasal lymphoma (*n* = 3), and mediastinal lymphoma (*n* = 1). The diagnosis of lymphoma was made by cytology of the lymph nodes or the mass lesions or by histopathological evaluation of tumour tissues. Polymerase chain reaction assay for clonal rearrangement in the T‐cell receptor gamma chain gene and in the immunoglobulin heavy chain gene were analysed using DNA samples extracted from the tumour cells or tissues in five cats. Immunohistochemistry for CD3 and CD20 were performed using tumour tissue samples in two cats. Based on the World Health Organization staging system for lymphoid tumours (Owen, 1980), seven cats were classified stage V lymphoma, while two cats could not be classified due to insufficient examinations. All cats had substage b lymphoma.

**TABLE 2:** Diagnosis and clinical staging in cats used in this study

Case No.	Diagnosis	Diagnostic methods	Clonality	Immunohistochemistry	Clinical stage
1	Large cell alimentary lymphoma	Cytology	TCRγ (‐), IgH (+)	Not examined	V (Manifestation in the blood)
2	Intermediate to large cell mediastinal lymphoma	Histopathology	TCRγ (‐), IgH (‐)	CD3 (+), CD20 (‐)	V (Pericardium involvement)
3	Large cell nasal lymphoma	Cytology	Not examined	Not examined	V (Kidney involvement)
4	Large cell nasal lymphoma	Cytology	TCRγ (‐), IgH (+)	Not examined	V (Kidney involvement)
5	Intermediate cell alimentary lymphoma	Histopathology	TCRγ (‐), IgH (‐)	CD3 (‐), CD20 (+)	Unknown
6	Large cell alimentary lymphoma	Cytology	TCRγ (+), IgH (‐)	Not examined	Unknown
7	Large cell alimentary lymphoma	Cytology	Not examined	Not examined	V (Manifestation in the blood)
8	Large cell alimentary lymphoma	Cytology	TCRγ (‐), IgH (+)	Not examined	V (Kidney involvement)
9	Large cell nasal lymphoma	Histopathology	Not examined	Not examined	V (Orbit involvement)

Abbreviation: CD, cluster of differentiation; IgH, immunoglobulin heavy chain gene; TCRγ, T cell receptor gamma chain gene.

Treatment condition in each cat is summarised in Table 3. Nimustine treatment was conducted in the primary therapy in two cats and in the rescue therapy in seven cats. All cats except one received prednisolone before initial nimustine administration. In the primary therapy group, one cat received l‐asparaginase (Leunase; Kyowa Kirin, Tokyo, Japan) at a dose of 400 K.U./kg three times before the initial nimustine administration, whereas the other cat received neither chemotherapy nor radiation therapy. The cat that received l‐asparaginase was included in the primary therapy group based on a previous study in which l‐asparaginase provided minimal benefit to cats with alimentary lymphoma (LeBlanc et al., 2007). In the rescue therapy group, five cats received chemotherapy including l‐asparaginase, cyclophosphamide hydrate (Endoxan; Shionogi & Co., Ltd; Osaka, Japan), doxorubicin (Adriamycin; Nippon Kayaku Co., Ltd.; Tokyo, Japan), and/or vincristine (Oncovin; Nippon Kayaku Co., Ltd.) before the initial nimustine administration. Three cats underwent radiation therapy.

**TABLE 3:** Treatment conditions, nimustine‐associated adverse events, and clinical outcomes in cats with lymphoma

		Treatment conditions of nimustine								
Case No.	Treatments before nimustine	Setting	Starting dose (mg/m^2^)	Number	Concurrent PSL therapy	Adverse events (grade)	Treatments after nimustine	PFS (days)	Current status	OS (days)
1	l‐asparaginase, Prednisolone	Primary	25	5	Yes	Neutropenia (1)	Dexamethasone, Doxorubicin, l‐asparaginase, Vincristine	688	Dead	745
2	None	Primary	30	11	Yes	Diarrhoea (1)	No treatment	274	Dead	275
3	CHOP therapy, Radiation therapy	Rescue	25	1	No	None	Cyclophosphamide, Dexamethasone, l‐asparaginase, Prednisolone	12	Dead	43
4	L‐CHOP therapy, Radiation therapy	Rescue	20	5	Yes	Lethargy (1), Thrombocytopenia (1)	Prednisolone	102	Dead	107
5	CHOP therapy	Rescue	20	2	Yes	Thrombocytopenia (1), Vomiting (1)	Dexamethasone, Prednisolone	106	Dead	110
6	L‐CHOP therapy	Rescue	20	1	Yes	None	Dexamethasone	9	Lost	>14
7	l‐asparaginase, Prednisolone, Vincristine	Rescue	25	11	Yes	Elevated ALT (2), Elevated AST (2), Diarrhoea (1)	No treatment	>671	Alive	>671
8	L‐CHOP therapy	Rescue	30	8	Yes	Elevated ALT (1), Thrombocytopenia (2)	Not performed	>148	Lost	>148
9	Prednisolone, Radiation therapy	Rescue	20	4	Yes	Allergic reaction (1), Vomiting (1)	CHOP therapy	65	Dead	91

Abbreviation: ALT, alanine aminotransferase; AST, aspartate aminotransferase; CHOP, Cyclophosphamide‐Hydroxyldaunorubicin (Doxorubicin)‐Oncovin (Vincristine)‐Prednisone; L‐CHOP, l‐asparaginase‐CHOP; OS, overall survival; PFS, progression‐free survival; PSL, prednisolone.

Nimustine was dissolved in water for injection and administered intravenously. The starting dose and dosing interval of nimustine were determined based on previous studies (Takahashi et al., 2014; Tamura et al., 2013). Median starting dose of nimustine was 25 mg/m^2^ (range: 20–30 mg/m^2^). In case 1, the dose was reduced by 5 mg/m^2^ after the fourth administration due to neutropenia. In cases 2, 4, and 8, the dose was increased by 5 mg/m^2^ after the sixth, second, and second administration, respectively. No dose changes were performed in the remaining cats. Dosing interval was set to 3 weeks. In case 1, the dosing interval had to be delayed every time due to neutropenia. Number of nimustine administrations were 5 and 11 in the primary therapy group and 1–11 (median: 4) in the rescue therapy group. All cats except one received concurrent daily administrations of oral prednisolone at median initial dose of 1.24 mg/kg (range: 0.29–2.44 mg/kg). In case 9, an allergic reaction was observed after the second administration of nimustine. Hence, pre‐treatment with d‐chlorpheniramine maleate (Polaramine; Takata Seiyaku Co., Ltd.; Tokyo, Japan) was performed before the third and subsequent administrations. Nimustine treatment was terminated in all cats except one (case 8) due to tumour progression (cases 3, 4, 6, and 9), remission (cases 1, 2, and 7), or request of the owner (case 5).

After termination of nimustine treatment, three cats received chemotherapy including l‐asparaginase, cyclophosphamide hydrate, doxorubicin, and/or vincristine. Five cats received steroid therapy including dexamethasone and/or prednisolone. Two cats received no treatment.

Adverse events associated with nimustine treatment were observed in seven cases (Table 3). Major adverse events included mild myelosuppression (*n* = 4) and gastrointestinal toxicity (*n* = 4). No severe adverse events (above grade 3) were observed in any of the cats. In case 1, nimustine was administered four times at a dose of 25 mg/m^2^. Neutropenia (grade 1) requiring dose delays was observed each time. However, this case did not exhibit any febrile neutropenia. Thrombocytopenia was observed in three cats. Two cats showed grade 1 only after the first administration of nimustine, whereas the remaining cat showed grade 1 or 2 after each administration of nimustine. None of the three cats required dose reduction or delayed medication. Four cats exhibited gastrointestinal symptoms (grade 1) including diarrhoea and vomiting. Hepatotoxicity was observed in two cats. One cat showed grade 1 or 2 after the third and subsequent administration of nimustine, whereas another cat showed grade 1 only after the sixth administration of nimustine. Lethargy (grade 1) was observed in one cat. One cat exhibited allergic reaction (grade 1) after the second administration of nimustine. Pre‐treatment with d‐chlorpheniramine maleate was performed before the third and subsequent administrations of nimustine, and no allergic reactions were observed.

Both the cats from the primary therapy group showed relapse during the study period. PFS were 688 and 274 days. Both died due to tumour progression during the study period. OS were 745 and 275 days. Among the seven cats from the rescue therapy group, five showed relapse during the study period. PFS were 9–671 days (median: 102 days). Four cats died during the study period. Cause of death in these cats was tumour progression. None of the cats were euthanised. One cat was still alive at the end of this study. Two cats did not return for the scheduled visit and thus, the clinical outcome was unknown. OS were 14–671 days (median: 109 days).

Previous studies have reported clinical outcomes in cats with lymphoma treated with lomustine in the primary therapy and in the rescue therapy. PFS and OS in cats with intermediate to large cell alimentary lymphomas treated with lomustine in the primary therapy were 31–1450 days (median: 132 days) and 4–1488 days (median: 108 days), respectively (Rau & Burgess, 2017). Additionally, PFS in cats with different types of small to large cell lymphomas treated with lomustine in the rescue therapy was 7–708 days (median: 39 days) (Dutelle et al., 2012). In the present study, PFS and OS in a cat (case 1) with large cell alimentary lymphoma treated with nimustine in the primary therapy were 688 days and 745 days, respectively. PFS in cats with different types of intermediate to large cell lymphomas treated with nimustine in the rescue therapy was 9–671 days (median: 102 days). The clinical outcomes in the cats with lymphoma treated with nimustine were similar to those treated with lomustine. Therefore, nimustine might be an alternative to lomustine in the treatment of feline lymphoma.

Lomustine has clinical benefits in the treatment of feline lymphoma and mast cell tumour (Dutelle et al., 2012; Rassnick et al., 2001, 2008; Rau & Burgess, 2017). However, it can cause severe myelosuppression including neutropenia and thrombocytopenia (Dutelle et al., 2012; Fan et al., 2002; Rassnick et al., 2001, 2008; Rau & Burgess, 2017; Saba et al., 2012). Other adverse effects associated with lomustine include gastrointestinal toxicity and hepatotoxicity (Dutelle et al., 2012; Musser et al., 2012; Rassnick et al., 2008). Rarely, cats receiving lomustine may be euthanized due to the severe toxicity (Fan et al., 2002; Saba et al., 2012). Although cats receiving nimustine exhibited myelosuppression, gastrointestinal toxicity, and hepatotoxicity similar to the ones receiving lomustine, no severe adverse events were observed. Since clinical outcomes in the cats with lymphoma treated with nimustine were similar to those treated with lomustine, nimustine might be a more useful agent than lomustine. However, the dose and dosing interval of nimustine in cats are controversial. Further studies are needed to optimize treatment condition of nimustine in tumour‐bearing cats.

One of the cats exhibited an allergic reaction after the second administration of nimustine. Similarly, an allergic reaction was reported in a cat with anaplastic oligodendroglioma after the seventh administration of nimustine (Tamura et al., 2013). Allergic reaction might be one of the notable adverse events related to nimustine use in cats. In the present study, pre‐treatment with d‐chlorpheniramine maleate was performed, and no allergic reactions were observed in the third and subsequent administrations of nimustine. Thus, pre‐treatment with d‐chlorpheniramine maleate might prevent this adverse event.

In addition to the lower toxicity, nimustine has other advantages over lomustine. Lomustine is available only in 10, 40, or 100 mg capsules for oral administration, as it is highly lipid soluble. Therefore, it would be difficult to adjust the dose for each case. Additionally, oral administration is difficult in cats. Since absorption of lomustine may be decreased in cats with gastrointestinal symptoms, it would also be difficult to achieve accurate dosing. In contrast to lomustine, nimustine is water‐soluble, and thus, it is available for intravenous or intraarterial administration. Hence, the aforementioned challenges related to lomustine administration are not encountered with the use of nimustine. This would make it possible to achieve accurate dosing.

In conclusion, nimustine was well tolerated and showed clinical outcomes similar to those observed with lomustine in cats with lymphoma. Our findings suggest that nimustine might be an alternative to lomustine in the treatment of feline lymphoma.

## AUTHOR CONTRIBUTIONS

**Kosei Sakai**: Conceptualization; data curation; formal analysis; funding acquisition; investigation; methodology; project administration; resources; software; supervision; validation; visualization; writing‐original draft; writing‐review and editing. **Shingo Hatoya**, **Masaru Furuya**, **Tomoyo Nabetani**, **Ryoji Kanegi** and **Shunsuke Shimamura**: Data curation; methodology; resources; writing‐review and editing. **Hiroyuki Tani** and **Terumasa Shimada**: Data curation; resources; writing‐review and editing

## CONFLICT OF INTEREST

The authors declare no conflict of interest.

## ETHICS STATEMNET

The authors confirm that the ethical policies of the journal, as noted on the journal's author guidelines page, have been adhered to. No ethical approval was required as this study did not include any experimentation on animals, and all data were generated from a part of daily clinical activities.

### PEER REVIEW

The peer review history for this article is available at https://publons.com/publon/10.1002/vms3.652

## Data Availability

The data that support the findings of this study are available from the corresponding author upon reasonable request.
